# The acquisition process of musical tonal schema: implications from connectionist modeling

**DOI:** 10.3389/fpsyg.2015.01348

**Published:** 2015-09-10

**Authors:** Rie Matsunaga, Pitoyo Hartono, Jun-ichi Abe

**Affiliations:** ^1^Department of Informatics, Shizuoka Institute of Science and Technology, ShizuokaJapan; ^2^School of Engineering, Chukyo University, NagoyaJapan; ^3^Department of Psychology, Hokkaido University, SapporoJapan

**Keywords:** computational modeling, connectionist model, schema acquisition, musical enculturation, scale and harmony

## Abstract

Using connectionist modeling, we address fundamental questions concerning the acquisition process of musical tonal schema of listeners. Compared to models of previous studies, our connectionist model (Learning Network for Tonal Schema, LeNTS) was better equipped to fulfill three basic requirements. Specifically, LeNTS was equipped with a learning mechanism, bound by culture-general properties, and trained by sufficient melody materials. When exposed to Western music, LeNTS acquired musical ‘scale’ sensitivity early and ‘harmony’ sensitivity later. The order of acquisition of scale and harmony sensitivities shown by LeNTS was consistent with the culture-specific acquisition order shown by musically westernized children. The implications of these results for the acquisition process of a tonal schema of listeners are as follows: (a) the acquisition process may entail small and incremental changes, rather than large and stage-like changes, in corresponding neural circuits; (b) the speed of schema acquisition may mainly depend on musical experiences rather than maturation; and (c) the learning principles of schema acquisition may be culturally invariant while the acquired tonal schemas are varied with exposed culture-specific music.

## Introduction

Musical abilities are largely shaped over time by experience with one’s culture-specific music in much the same way that human linguistic skills are determined by culture-specific language experiences (Hannon and Trainor, 2007). The considerable contributions of the acquired and culture-specific experiences should hold for musical skills related to “tonality” which are fundamental to music. Many studies have shown that cognitive representations of musical pitch structures such as tonality, scales, and modes, clearly change as a function of exposure to culture-specific musical conventions (Kessler et al., 1984; Trainor and Trehub, 1992, 1994). This raises a fundamental question: How does the human brain acquire a culture-specific tonal schema through mere exposure to music? Our aim is to address this question using an approach based on connectionist (or artificial neural network) modeling.

### Tonal Schema Acquisition

A number of studies addressing the acquisition of tonal schema have focused on children who have grown up in a culture of Western music. Findings of these studies clearly indicate that the level of tonal schema held by adults is not achieved in children until late in their development (Trainor and Hannon, 2013, for a review). Trainor and Trehub (1992) show that unlike adults, young infants are not sensitive to differences between the notes belonging to a musical scale of one’s cultures (i.e., in-scale tones; e.g., C, D, E, F, G, A, B in C major of Western diatonic scale) and those which do not (i.e., out-of-scale tones; e.g., C#, D#, F#, G#, A# in C major of Western diatonic scale). In their study, Trainor and Trehub (1992) presented adults and 8-month-old infants with 10-tone melodies and measured their ability to detect occasional tone changes. Adults showed significantly higher detection rates of changes to tones that did not belong to the melodic key compared to tones within the key; by contrast, infants of 8 months showed that, although they detected both changes, their detection rates did not significantly differ between both changes. This result suggests that infants as young as 8 months have not yet acquired the schema of scale membership for the Western diatonic scale. However, by at least the age of 4 years, a scale schema appears to be in place. Four- and 5-year-old children can differentiate between tone sequences that conform to the Western diatonic scale and those that do not (Trehub et al., 1986); moreover, as with adults, they perform better at detecting out-of-key changes in a melody than within-key changes (Trainor and Trehub, 1994; Corrigall and Trainor, 2010, 2014).

From the historical and anthropological views, the modern Western music has been unique in harmony. Several studies have argued that musically westernized listeners develop a scale schema early in development and then a harmonic schema (to be exact, a schema of the implied harmonic function) at a later point (Costa-Giomi, 2003; Schellenberg et al., 2005; Corrigall and Trainor, 2010, 2014). For example, Trainor and Trehub (1994) found that although 5-year-old children were sensitive to out-of-key changes only, 7-year-old children were sensitive to not only out-of-key changes but also out-of-harmony changes in Western melodies. Using the probe-tone technique that involves presenting a tonal context followed by individual tones that must be rated for how well they fit within the established tonal context, Krumhansl and Keil (1982) found that younger children showed only distinctions between in-scale and out-of-scale tones, whereas older children distinguished triad scale tones from non-triad scale tones. The perception of triad scale tones is, in part, fundamental to sense of the implied harmony. In short, a harmonic schema develops relatively late; further, it is likely that it does not reach the level of adults until a child’s early teenage years.

Enculturation to the Western tonal system follows a clear developmental trajectory in which the acquisition of scale schema is followed by the acquisition of harmony schema. Some researchers hypothesize that the order of acquisition of scale and harmony is related to the degree of culture universality (Hannon and Trainor, 2007; Trainor and Unrau, 2012). Although we do not disagree with this idea, we believe that this order of acquisition can be explained with a relatively more straightforward concept based on characteristics related to constraints of scale system and harmony system. At a basic level, a scale system is characterized by constraints of scale memberships (i.e., the notes belonging in the scale), and a harmony system is characterized by constraints of the scale system. Thus, a scale system underlies a harmony system, but the converse is not true. From this perspective, then, it is not surprising that an individual’s acquisition of scale schema begins *before* the acquisition of harmonic schema and that the acquisition period for harmonic schema is longer than the acquisition period for scale schema. Furthermore, this view can offer answers to relevant questions, such as why scales are found in virtually all cultures but certain concepts of harmony are specific to Western music; or the question of why, historically, the establishment of scales began before the establishment of harmony in Western music.

### Tonal Schema Acquisition and Connectionist Networks

The present study uses a computational modeling approach with potential for explaining the mechanism responsible for tonal schema acquisition. In general, the main focus of most cognitive scientists using this approach has been to capture essential cognitive functions that emerge from vast ensembles of physiological neuronal elements in the brain (e.g., Elman et al., 1996; O’Reilly and Munakata, 2000; Munakata and McClelland, 2003). In line with this tradition, the main focus of the present study is to understand the functionality of tonal schema acquisition. This is distinguished from a goal of understanding the biological origins of functionality. In other words, we seek to develop a network that shows common cognitive functions with the human brain, but we do not constrain the implementation of this network to reflect current biological speculations about brain operations.

To clarify these points, let us outline the basic requirements that a viable connectionist model of the acquisition of tonal schema is expected to fulfill.

• **Requirement 1:** The model should be capable of learning a tonal schema by mere exposure to music. This requirement is justified by much evidence which shows, as in language acquisition, that listeners have an ability to acquire a tonal schema during passive exposure to music (Trainor and Trehub, 1992, 1994).
• **Requirement 2:** The model should be bound by assumptions of culture-general property. In other words, culture-specific assumptions should be unnecessary or minimized. The requirement is also justified by evidence which shows, as in language acquisition, that together with innate constraints, infants are basically born culture free with respect to tonal schema acquisition (Lynch et al., 1990; Schellenberg and Trehub, 1999).
• **Requirement 3:** The model should be trained with musical materials that directly reflect the musical environment experienced by general listeners in a culture as much as possible. Practically speaking, this last requirement is challenging because the actual states of musical environments are not clear (i.e., questions of what, when, how much music a majority of listeners have encountered are hard to answer). Therefore, a possible strategy for the selection of music materials for the network training is to utilize the existing music that are familiar to most listeners of a culture. In the case of use of the existing music, we think that monophonic melodies (tone sequences) are preferred to polyphonic melodies and chord sequences. The reason for this preference is that monophonic melodies are common to virtually all known musical cultures except for the modern Western music. In addition, in the sense that monophonic melodies established earlier than polyphonic melodies in even Western music culture (Shibata, 1967), monophonic melodies can be considered to be primitive ones. Moreover, infants and children, who are learning a tonal schema in progress, have quite a lot of opportunities for exposure to monophonic melodies through, for example, parents’ singing to infants/children.

In the following paragraphs we review previous studies that have developed artificial connectionist models in the field of tonality perception. Of particular concern is the degree to which such approaches to connectionists modeling have met the preceding requirements for a viable model.

Studies using connectionist models have sought to explore the mechanism of tonal schema of musically westernized listeners (Bharucha, 1987; Griffith, 1999; Tillmann et al., 2000; Toiviainen and Krumhansl, 2003; Large, 2010). The pioneering connectionist model (MUSACT) of Bharucha (1987) addressed tonal perception of Western listeners. In this model network units (or neurons) were organized in three layers corresponding, respectively, to tone chromas, chords, and keys. Connection weights were based on a *prior* setting following certain guidelines (music theory, psychological evidence). Specifically, in MUSACT each of 12 tone chroma units was connected to three major and three minor chords, of which that chroma was a component; analogously, each chord unit was connected to three major key units representing the keys of which it was a member. MUSACT simulated a psychological phenomenon in which, for example, human listeners feel the heightened expectancy of F major key rather than F# major key, when hearing a C major chord. In other words, the unit for F major key was activated to a greater degree than the unit for F# major key when a chord consisting of C, E, and G was given to MUSACT. A subsequent study reported that MUSACT was successful in explaining some features of listeners’ expectancy for a sequence of chords (Bigand et al., 1999). However, MUSACT did not incorporate a learning mechanism; therefore in this point, MUSACT, like several symbolic computational models (e.g., Yoshino and Abe, 2004; Temperly, 2008), is unable to account for the acquisition process of tonal schema.

A different connectionist model, which has the learning mechanism, was proposed by Tillmann et al. (2000). The network model was based on a self-organizing map (SOM). The SOM is trained in an unsupervised manner to map high dimensional data into low dimensional space while preserving the data’s topological order (topological preserving characteristics of SOM ensure that data points similar in original high dimensional space are mapped into close neighboring areas in the visualizable two or three-dimensional map). In this SOM, units were organized in three layers corresponding to tone chromas, chords, and keys. Training materials for the SOM comprised 10 kinds of seven-chord sequences, terminating in either a V–I or IV–I progression of major key contexts. After training, the SOM derived a topological map of major keys in the third layer. Tillmann et al. (2000) demonstrated that the trained SOM was run through a range of experiments from previous researches, and it simulated a variety of psychological phenomena, such as chord priming and effects of circle-of-fifths on perception of key modulation.

In light of the three (previously stipulated) basic requirements, the network model of Tillmann et al. (2000) falls short with respect to two of these requirements. First, their training materials did not directly reflect music that most listeners encounter in daily life. Rather, they employed very brief chord sequences (i.e., a sequence of only seven chords) as melodic materials for training. Also, they did not use any melodic training materials with minor keys. Second, their model was bound by an assumption of culture-specific property. That is, the intermediate network layer of their model was designed to represent harmonic processing, in spite of the fact that harmonic processing is unique to listeners of Western music.

A few other studies have indirectly engaged in simulations of tonal schema learning process. For example, Griffith (1999) constructed a SOM network, in which units were organized in two layers corresponding to tone chromas and keys. His network was trained with the distribution information of frequency of occurrence of tone chromas. Another SOM model was constructed by Toiviainen and Krumhansl (2003) in which units were organized in two layers corresponding to tone chromas and keys. To develop a map of musical keys, the network was trained by listeners’ tone ratings in probe-tone profiles for 24 major and minor keys (Krumhansl and Kessler, 1982). Networks of both Griffith and of Troiviainen and Krumhansl fulfill two of the three basic requirements listed above, i.e., they provided a learning mechanism, and they did not have prior assumptions of culture-specific properties. However, the melodic training materials in both studies did not directly reflect music environment that the general Western music experience.

A different approach to this topic is found in the connectionist model proposed by Large (2010). In this account, the learning mechanism is grounded in neurophysiological evidence and it avoids assumptions of culture-specific property. However, details of melodic training materials for this network remain unclear.

In summary, all pertinent connectionist networks appear to fall short in at least one of the three basic requirements. Moreover, none have reported detailed observations on the learning process of tonal schema by the connectionist network. Thus, one aim of the present study is to build a connectionist network that essentially meets all three basic requirements outlined above. A second aim is to assess how well this network acquires tonal schema when exposed, over time, to Western tonal music.

## Simulating Western Tonal Schema Acquisition with LeNTS

The present study proposes a connectionist network (Learning Network for Tonal Schema, or LeNTS) of tonal schema acquisition. This network is designed to fulfill the three basic requirements. To meet the first requirement, LeNTS was designed to have a learning function. Specifically, it had a supervised learning algorithm, i.e., the backpropagation learning algorithm (Rumelhart et al., 1986). This means that, during the learning process, LeNTS learns associations between input data (i.e., a stimulus melody) and its corresponding teacher signal (i.e., a key name that human listeners perceive). Here it should be noted that, like many psychologists using the connectionist model with a supervised learning algorithm, we do not assume that in the general listeners’ environment there is an “explicit teacher signal” that tells the musical key of a given melody. Nevertheless, this does not exclude the possibility of the presence of “implicit” teacher signal. Considering how listeners accomplish a tonal schema learning, we think it is reasonable to assume the presence of some unknown “implicit” teacher signals. However, at present, no one knows the nature of the “implicit” teacher signal, and no one offers the methodology that deals with “implicit” teacher signals. More generally, psychology has never empirically and theoretically elucidated how human learns a schema. Under such circumstance, an approach of connectionist model with learning functions is the only methodological approach that would be able to offer substantial implications for the unsolved question. In short, we do not intend to justify our strategy in which the teacher signals are “explicitly” given to the connectionist model; but instead, our intention is that, at the present stage, the approach of doing simulations by the explicit teacher signals is much more constructive than doing nothing.

Next, to meet the second requirement, we designed hidden and input layers of LeNTS. Regarding the hidden (intermediate) layer, we designed not to make any special assumptions in it. When no assumption can be made, it is common to arrive at a number of hidden layers by trial and error. We therefore decided to determine the number of hidden layers on the basis of the results of a preliminary study in which networks with different numbers of hidden layers were compared (described later). Regarding the input layer, we designed it to represent a culture-general property only. Specifically, we represented 12 tone chromas per octave in the input layer. This is because 12 per octave can be considered to be a culture-universal property. This idea is supported by the fact that many non-Western music cultures have inclusive scales approximately equivalent to Western 12-chromatic scale (cf. Kuttner, 1975; Deva, 1981; Burns and Ward, 1982; Koizumi, 1984 for non-Western music^1^); in addition, this idea is also supported by empirical evidence that listeners have difficulties in precisely identifying or labeling a narrow pitch interval (e.g., a quarter tone) less than a semitone interval (defined by a ratio of two frequencies, as 1.00:1.06) (Burns and Ward, 1978).

By contrast to the settings of hidden and input layers, for the setting of output layer we had no choice but to bring a culture-specific assumption. If our cognitive model could automatically emerge output units (i.e., perceptual categories that listeners finally acquire) by the mere exposure to the inputted culture-specific stimulus data, we would not need to bring the culture-specific assumption. However, in fact, at present, no researcher has proposed and acquired such ideal methodology. And, needless to say, no researcher has ever achieved understanding how perceptual categories emerge in the brain. Therefore, we had to pre-design the output layer to represent culture-specific tonal categories (i.e., output units). Specifically, in the output layer, we represented 24 tonal categories (i.e., 24 key-name categories = 12 tonal centers × 2 major/minor modes) derived from Western diatonic scale. This is because the present study focuses on the general children (i.e., those who are learning a tonal schema in progress) of Western music culture. It is very clear that, for the general children in the Western music culture, the exposure to music of Western diatonic scale is overwhelmingly majority in their daily life.

Finally, to meet the third requirement, we trained LeNTS with existing music materials that are familiar to the general children in the musical culture of Western. We selected 356 kinds of Western melodies as the training materials from many different kinds of children’s songbooks (e.g., music textbooks often used in elementary schools). In the songbooks, each of the 356 melodies was denoted as a sequence of tones (i.e., monophonic melodies or melody lines of homophonies). Of the 356 melodies, 315 and 41 were denoted as major keys and minor keys, respectively, in the musical scores. That is, in the training materials of this study, melodies with ‘major’ keys were larger in number than those with ‘minor’ keys. This distribution should be essentially consistent with the musical experiences of most Western listeners. After selecting the 356 melodic training materials, we extracted the information of ‘sequence of tone chromas’ from each melodic material following the format of input layer of LeNTS.

In this study, LeNTS was tested with novel set of tone sequences (i.e., not used in training) after every 5,000 epochs of training—in this study one each melody appeared once in one training epoch. This was to trace the network’s process of ability changes in tonal processing. Training was set to finish at epoch 50,000; accordingly, testing was implemented a total of 10 times. For each test, activations of 24 key units (12 major key units and 12 minor key units) in the output layer were read out to evaluate keys identified by LeNTS.

Tone sequences for network testing were prepared on the basis of findings of previous studies of tonal schema acquisition of Western music by the general children. As noted earlier, many studies have indicated that the Western children acquire musical scale schema early in development and then harmony schema at a later point (e.g., Trainor and Hannon, 2013). With this in mind, we created testing materials following two criteria: first, all tones comprising a sequence can be interpreted as scale tones of the Western diatonic scale. Second, tone sequences can validly imply the Western tonal harmony. If LeNTS can acquire the schema of Western diatonic scale, its learning profile should manifest different performances between tone sequences that conform to the Western diatonic scale and those that do not. Likewise, if LeNTS acquires the schema of Western harmony, then the network’s learning profiles will differ for tone sequences that can be perceived as the implied harmony and those that cannot. Based on this rationale, we sought to identify the respective times of asymptotic learning for scale and harmony schemas.

### Methods

#### Connectionist Network

**FIGURE 1:**
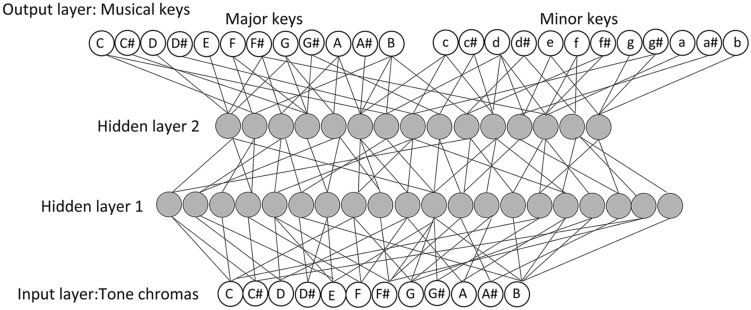
**The architecture of Learning Network for Tonal Schema (LeNTS).** The input layer represents 12 tone chromas. The output layer represents 24 keys (12 major keys and 12 minor keys). The number of units in each hidden layer is determined arbitrarily (45 units in hidden layer 1, 30 units in hidden layer 2).

The network architecture used in this study was a Multilayered Perceptron (Rumelhart et al., 1986; **Figure 1**). This architecture was implemented in the MATLAB. The input layer represented 12 tone chromas. Inputs to the network were the melodic materials (mentioned above); to be specific, these melodies are sequences of tone chromas. Several studies have indicated that differences in the temporal ordering of tones in given melodies (e.g., C–G–E–A–D–B, D–B–C–E–A–G, etc.) influence tonality perception (Matsunaga and Abe, 2005). Accordingly, we prepared an input processing system that was able to treat differences in the temporal ordering of tones in the prepared melody materials as different inputs. Specifically, the constituent tone chromas, resulting from the input of any melody, were coded by their specific serial positions (e.g., the first position, the second position) in the melody. Among the melody materials we prepared, the longest melody contained 186 tones; hence, the input processing system of LeNTS was designed to be able to handle an inputted melody up to 186 tones (in length). In this fashion, LeNTS allows for differentiation of temporal orderings of tones that determine different melodies.

The output layer of LeNTS represented 24 keys (12 major keys, and 12 minor keys; **Figure 1**). During the training process, the correct key (i.e., the key most listeners will perceive for a tone sequence; the key denoted in the musical score of children’s songbooks) was presented as a teacher signal expressed as 24 bits of {0, 1} binary string. The teacher signal contained a single “1” which position in the 24-bit string indicated the correct key for the inputted training melody materials. In conformity with the teacher signals, each output unit was associated with a particular key, while its output value indicated identifiable (presence) or non-identifiable (absence) as that key.

Regarding the hidden layers, two layers were created on the basis of the results of the preliminary study (Appendix). Following methods used in prior studies (e.g., Ueno et al., 2011; McClelland, 2013), the number of units in each hidden layer was determined arbitrarily (45 units in the first hidden layer, 30 units in the second hidden layer).

The activation function for all the hidden units and output units was the Hyperbolid Tangent Sigmoid function, f(x), defined as follows:

(1) $$\text{f}(\text{x})\text{=}\frac{\exp\;(\text{x})\text{-}\exp\;(\text{-x})}{\exp\;(\text{x})\text{+}\exp\;(\text{-x})}\;$$

Because f(x) ∈ (-1, 1), during the training, values of output units, *O*^i^, were binarized as follows

(2) $$y^{i}=\left\{\begin{array}{cc} 1 & forO^{i}>0\\ 0 & otherwise \end{array}\right\}\;\left(2\right)$$

$$-1<O^{i}<1$$

Here, *y*^i^is the binarized value of the i-th output unit and *O*^i^ is its original value.

#### Melody Materials: Training and Testing

##### Training materials

For LeNTS, training materials involved 356 melodies that conformed to Western diatonic scales and were generally familiar to the general Western children. The 356 melodies were selected from various kinds of children’s songbooks (e.g., “Atarashii ongaku (Tokyo Shoseki),” “Ongaku no okurimono (Kyoiku Shuppan),” and so on), and then they were modified to monophonic isochronous sequences of tone chromas. According to these books, most Western listeners would clearly perceive each melody in being in one stable key. Therefore, we identified a key denoted in the musical score as the “correct key (i.e., a key most listeners typically perceive)” in this study. This is in line with the view that most listeners basically perceive the same musical key as denoted in the score (Abe, 2008). The selected 356 melodies consisted of 315 melodies with major keys and 41 with minor keys. The total number of tones per melody differed from 17 to 186 tones. The frequency distributions of in-scale and out-of-scale tones for the 356 melodies and those for a large sample of Western melodies [data reported by Krumhansl (1990); i.e., these data reflect a combination of data from Youngblood (1958) with those of Knopoff and Hutchinson (1983)] were very similar [*r*(10) = 0.99, *p* < 0.01 for the major key; *r*(10) = 0.93, *p* < 0.01 for the minor key]. In providing the training melodies to LeNTS, these melodies were actually transposed to 12 different keys. That is, if the original key of a melody was a major key, the melody was transposed to all 12 major keys. Likewise, the melody was transposed to all 12 minor keys if the original key was a minor key. As a result, the network was trained with a total of 4,272 melodies in 1 epoch (315 melodies × 12 major keys + 41 melodies × 12 minor keys). By the manipulation of transposition, all major keys (or all minor keys) shared the same distributional pattern of in-scale and out-of-scale tones. Hence, it could be assumed that LeNTS, which was trained to the 4,272 melodies, did not have learnability biases to specific keys within each mode.

##### Testing materials

Testing melody materials met two criteria. The first criterion was that all constituent tones of a sequence were interpretable as in-scale tones of the Western diatonic scale; the second criterion was that a tone sequence could be perceived as implying the standard harmony of Western tonal music. In this study, we prepared three groups of tone sequences as input data for testing: (a) One group of tone sequences conformed to the Western diatonic scale and was designed to imply a regular harmonic progression; (b) a second group of sequences conformed to the Western diatonic scale but did not imply a regular harmonic progression; (c) a third group of sequences conformed neither to the Western diatonic scale nor to an implied regular harmonic progression. From the viewpoint of scale, tone sequences of both (a) and (b) groups can be characterized as comprising all in-scale tones of a Western diatonic scale, while sequences of (c) group can be characterized as including out-of-scale tones. From the viewpoint of harmony, tone sequences of (a) group can be characterized as implying a regular harmonic progression while sequences of both (b) and (c) groups can not. On the basis of these characteristics, the (a), (b), and (c) groups constituted the three major conditions central to the present study and labeled as: *in-scale/regular-harmony*, *in-scale/irregular-harmony*, and *out-of-scale/irregular-harmony* conditions, respectively.

**FIGURE 2:**
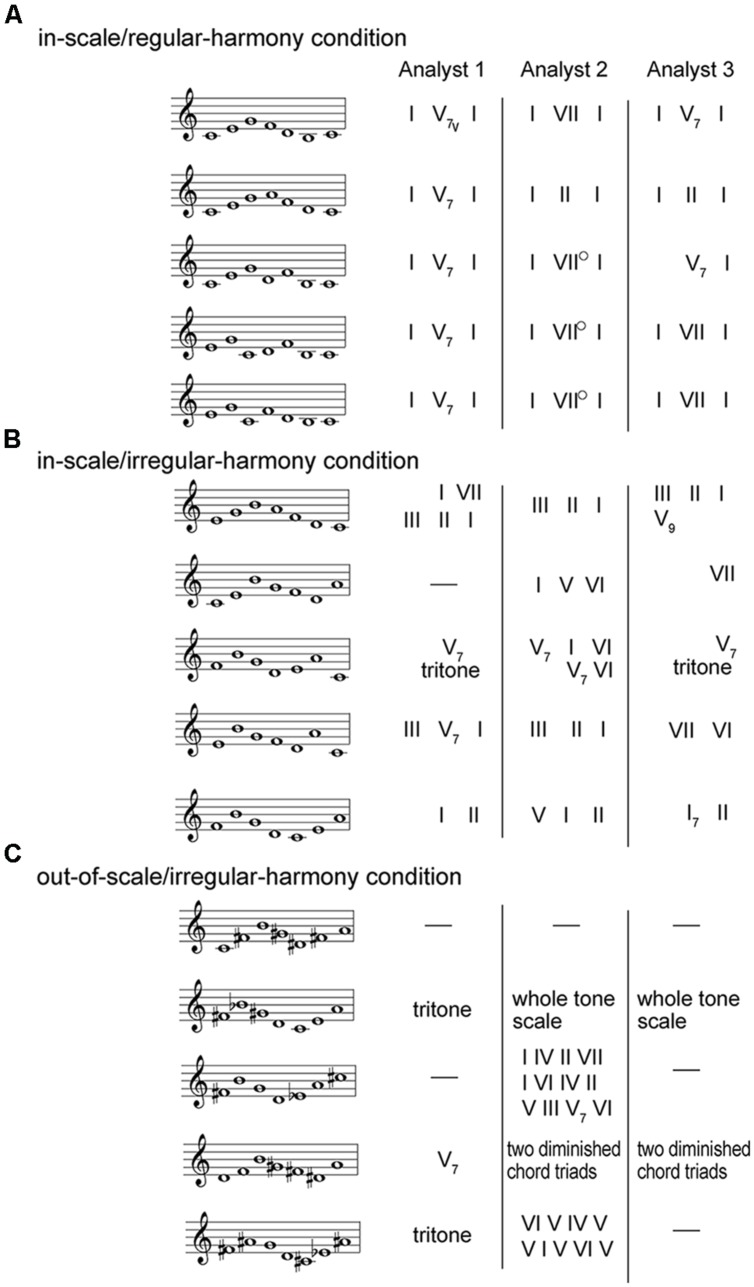
**Fifteen kinds of testing melody materials. (A)** The in-scale/regular-harmony condition contained five tone sequences, all conforming to the Western diatonic scale and implying a regular harmonic progression. **(B)** The in-scale/irregular-harmony condition contained five other tone sequences, all conforming to the Western diatonic scale but lacking clear implications of harmonic progressions. **(C)** The out-of-scale/irregular-harmony condition contained five different sequences, all conforming neither to the diatonic scale nor did they implicate a regular progression. All testing materials were selected from materials of Cuddy et al. (1981). The musical scores were denoted in the movable doh system. In the study of Cuddy et al. (1981), three professional musicians (Analyst 1–3) identified both keys and the harmonic progressions. Harmonic analyses of the three musicians appear in the right hand section. According to Cuddy et al. (1981), Analyst 2 commented that, where VII was noted, he/she usually meant to imply harmony at the dominant.

The above three conditions contained tone sequences that were drawn from materials used in Cuddy et al.’s (1981) study (**Figure 2**). They presented brief tone sequences (all isochronous) to three professional musicians who had to identify keys and the harmonic progression(s) implied by each sequence. On the basis of a summary of results of Cuddy et al. (1981), we selected three groups of tone sequences for testing. Specifically, the in-scale/regular-harmony condition contained five tone sequences (**Figure 2A**), all conforming to the Western diatonic scale and implying a regular harmonic progression (i.e., I–V–I or I–IV–I). The in-scale/irregular-harmony condition contained five other tone sequences (**Figure 2B**), all conforming to the Western diatonic scale but lacking clear implications of harmonic progressions. The out-of-scale/irregular-harmony condition contained five different sequences (**Figure 2C**) that conformed neither to the diatonic scale nor did they implicate a regular progression. When these testing sequences were presented to LeNTS, *doh*(s) in the musical score of Cuddy et al. (1981) were positioned as tone chroma C. Therefore, listeners should typically perceive C major as the “correct” key for all the sequences^2^ consistent with findings reported by Cuddy et al. (1981).

#### Procedure

##### Training procedure

In one epoch, LeNTS was trained with 4,272 kinds of the inputted melody materials (tone chroma sequences) and their corresponding teacher signals (musical keys). Processing inputs during training followed the standard procedure for backpropagation learning, where LeNTS attempted to approximate teacher signals for the inputs. LeNTS was trained for 50,000 epochs.

##### Testing procedure

The first testing procedure was implemented after 5,000 training epochs. Subsequent testing occurred every 5,000 epochs until the number of training epochs reached 50,000, creating 10 test times. In each testing, LeNTS was provided with five sequences for each of three major conditions. LeNTS calculated activations of 24 output units for a given input sequence. No connection adjustment occurred during testing.

### Simulation Results

Ten different runs of LeNTS were conducted. For each run, LeNTS was initialized with random connection weights. Simulation results showed that, unlike the nine other runes, one run failed to make progress in training. The training process of LeNTS was based on gradient descent with random initialization, thus it might be occasionally trapped in a very shallow local minima immediately after the training process was started, as in this case. We did not cherry-pick the good run; however, for analysis, we had to pick runs that were, to some extent, able to be trained (regardless of the training performance). For this reason, we excluded the anomaly run. That is, simulation results averaged across the remaining nine runs are reported below.

#### Training

**FIGURE 3:**
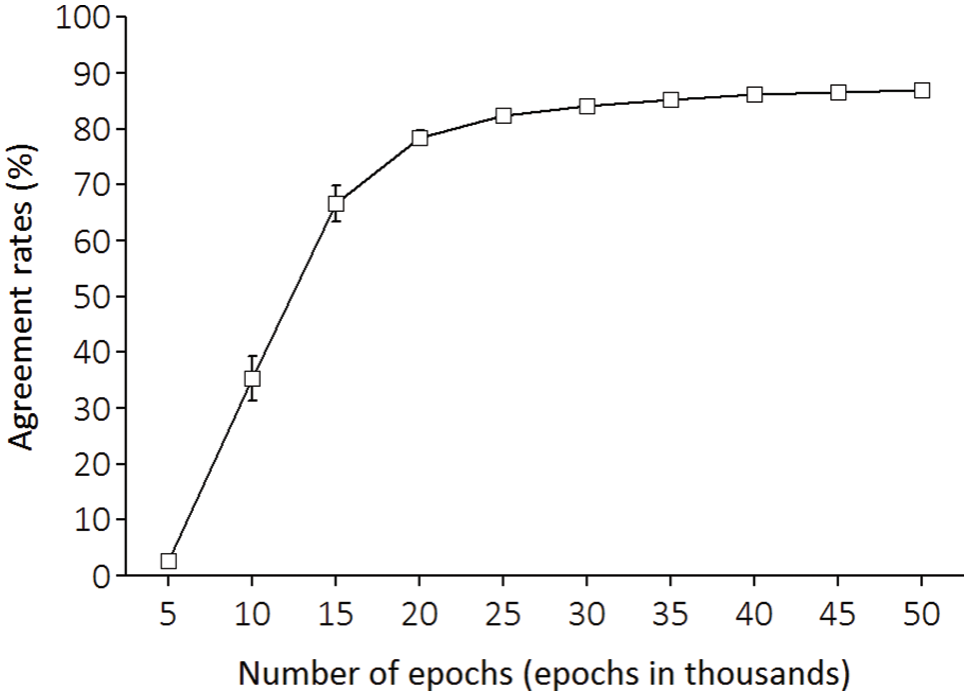
**For the training melody materials, agreement rates between keys identified by the network and correct keys.** The x-axis shows the number of training epochs.

After every 5,000 epochs, we repeated the input of the same set of training melodies to LeNTS, and LeNTS produced an activation pattern of the 24 output units for each training melody. At this time, we identified LeNTS’ musical key as determined by the output unit with the largest activation value computed by this model. Then, we calculated how much this key, identified by LeNTS, agreed with the key notated in original musical score (from the songbooks for children) for this melody which functioned as the teacher signal. As **Figure 3** illustrates, the network learning improved over the course of training. In the final training epoch (epoch 50,000), LeNTS reached an agreement rate level of 87%.

#### Testing

**FIGURE 4:**
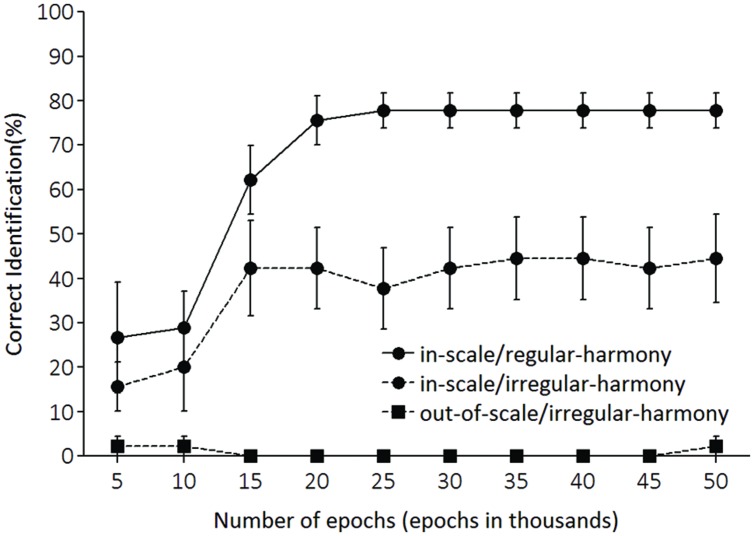
**Percentages of correct key identification for each testing condition.** The x-axis shows the number of training epochs.

**FIGURE 5:**
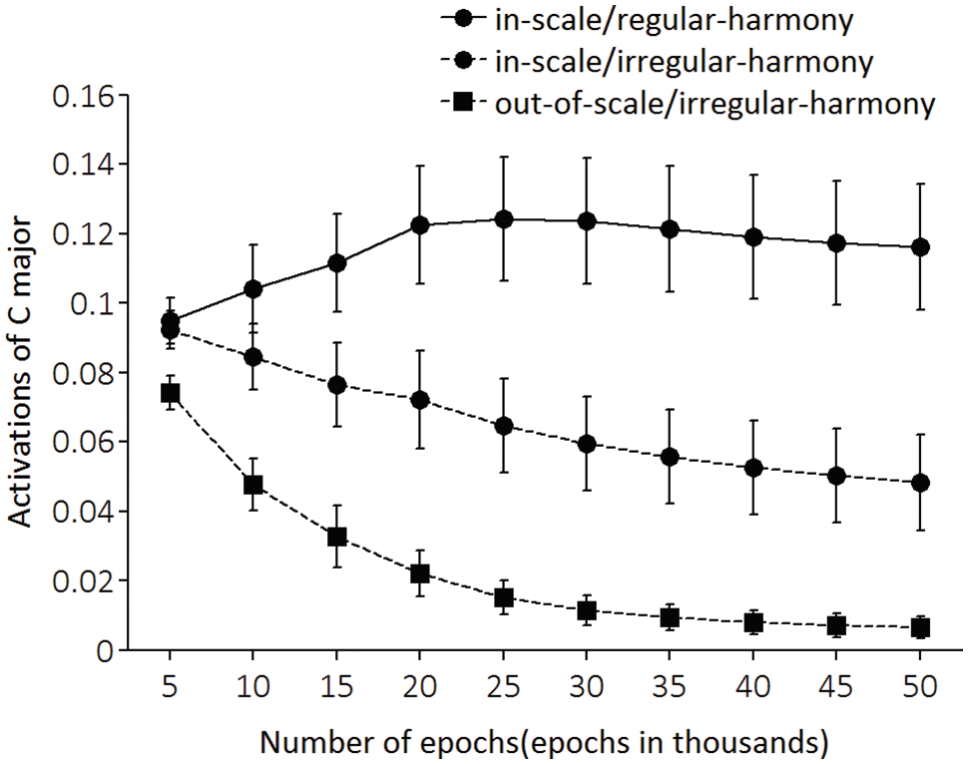
**Activations of C major unit (i.e., a correct key unit).** The activation values of each output unit was set to range from –1 to +1, but values were normalized to a range from 0 to +1 for readability. The x-axis shows the number of training epochs.

Test performances in each of the three conditions (in-scale/regular-harmony, in-scale/irregular-harmony, and out-of-scale/irregular-harmony conditions) were assessed every 5,000 epochs. As with training, LeNTS produced an activation pattern of the 24 output units for each test tone sequence, and a key associated with the output unit having the largest activation value was adopted as the key identified by LeNTS for each input sequence—results of Cuddy et al. (1981) imply that all sequences will be heard in C major. For each sequence, a network performance was deemed correct if the LeNTS produced the largest activation to the output unit of C major. **Figure 4** shows the correct percentages for each condition. **Figure 5** shows activation values of C major (i.e., a psychologically correct key) for each condition.

Correct key identification data were submitted to a two-way 3 (condition) × 10 (epoch) repeated measures analysis of variance (ANOVA). This analysis revealed a significant main effect of epoch [*F*(9,72) = 10.46, *p* < 0.001], condition [*F*(2,16) = 64.97, *p* < 0.001], and a significant interaction of condition with epoch [*F*(18,144) = 7.39, *p* < 0.001]. Furthermore, significant simple main effects of condition at epoch were observed (**Table 1**; alpha level = 0.05 in simple main effect tests; the adjusted alpha level = 0.05 in pair-wise comparisons). On epoch 5,000 [i.e., epoch 5 (epoch in 1000s) in **Figure 4**], the correct percentage for the in-scale/regular-harmony condition (27%) was significantly higher than that for the out-of-scale/irregular-harmony condition (2%), but no significant differences were found in other comparisons. On epoch 10,000, there were significant differences in one comparison between in-scale/regular-harmony and out-of-scale/irregular-harmony conditions, and another comparison between in-scale/irregular-harmony and out-of-scale/irregular-harmony conditions. These results indicate that the trained LeNTS was significantly better at identifying correct keys for the tone sequences conforming to the Western diatonic scale than for the sequences that did not conform. But, the percentages of correct identification remained low (approximately 20–30 %) even in the in-scale/regular-harmony and in-scale/irregular-harmony conditions.

**Table 1:** Results of pair-wise comparisons for correct key identification data.

			Pair-wise comparisons		
Epoch (in 1000s)	*F*(2,160)	*p*	In-scale/regular-harmony vs. in-scale/irregular-harmony	In-scale/regular-harmony vs. out-of-scale/irregular-harmony	In-scale/irregular-harmony vs. out-of-scale/irregular-harmony
5	4.831	<0.01	ns	*	ns
10	5.946	<0.01	ns	*	*
15	32.541	<0.001	*	*	*
20	46.238	<0.001	*	*	*
25	48.786	<0.001	*	*	*
30	48.892	<0.001	*	*	*
35	49.104	<0.001	*	*	*
40	49.104	<0.001	*	*	*
45	48.892	<0.001	*	*	*
50	46.238	<0.001	*	*	*

*p < 0.05.

Network performance improved with more training. On epoch 15,000, correct percentages in key identification increased markedly in some conditions. Correct identifications were highest for the in-scale/regular-harmony condition (62%), and next highest for the in-scale/irregular-harmony condition (42%). However, performance was very poor for the out-of-scale/irregular-harmony condition (0%). These results indicate that LeNTS was significantly better at identifying correct keys for tone sequences with regular-harmony than for sequences characterized by irregular-harmony. In the largest number of epochs (epoch 50,000), correct percentages approximated 80% for the in-scale/regular-harmony condition, whereas accuracy leveled off around 40% for the in-scale /irregular-harmony condition. And, the out-of-scale/irregular-harmony condition remained 0% throughout the training.

Data of C major activation (**Figure 5**) were submitted to a two-way 3 (condition) × 10 (epoch) repeated measures ANOVA. The analysis revealed a significant main effect of epoch [*F*(9,72) = 6.77, *p* < 0.001], condition [*F*(2,16) = 71.92, *p* < 0.001], and a significant interaction of condition with epoch [*F*(18,144) = 16.80, *p* < 0.001]. Furthermore, results of simple main effects of condition at epoch (**Table 2**) were very similar to those of correct key identification data. On epoch 10,000 [i.e., epoch 10 (epoch in 1000s) in **Figure 5**], there were significant differences in one pair-wise comparison between in-scale/regular-harmony and out-of-scale/irregular-harmony conditions. Also the pair-wise comparison of the in-scale/irregular-harmony condition with that of out-of-scale/irregular-harmony was significant. From epoch 15,000 to epoch 50,000, the activations of C major were highest for the in-scale/regular-harmony condition, next highest for the in-scale/irregular-harmony condition, and lowest for the out-of-scale/irregular-harmony condition. All the latter pair-wise comparisons were also statistically significant.

**Table 2:** Results of pair-wise comparisons for activations of C major unit (i.e., correct key unit).

			Pair-wise comparisons		
Epoch (in 1000s)	*F*(2,160)	*p*	In-scale/regular-harmony vs. in-scale/irregular-harmony	In-scale/regular-harmony vs. out-of-scale/irregular-harmony	In-scale/irregular-harmony vs. out-of-scale/irregular-harmony
5	2.903	0.0578	ns	ns	ns
10	18.870	<0.001	ns	*	*
15	35.805	<0.001	*	*	*
20	57.661	<0.001	*	*	*
25	68.406	<0.001	*	*	*
30	72.262	<0.001	*	*	*
35	72.551	<0.001	*	*	*
40	71.448	<0.001	*	*	*
45	70.558	<0.001	*	*	*
50	70.116	<0.001	*	*	*

*p < 0.05.

### Discussion

Relative to the previous studies mentioned in Introduction (Bharucha, 1987; Griffith, 1999; Tillmann et al., 2000; Toiviainen and Krumhansl, 2003; Large, 2010), our connectionist model was more successful in meeting three basic requirements needed for a viable human cognitive model. We examined how LeNTS developed tonal processing abilities throughout training of Western melodies.

Overall, the simulation results show that LeNTS can learn a relevant tonal schema. On epoch 10,000, LeNTS was sensitive to differences between tone sequences consisting of all in-scale tones and sequences including out-of-scale tones; but, at that time, it did not yet distinguish differences in the implied Western harmony. However, on epoch 15,000, LeNTS showed not only sensitivity to differences between tone sequences consisting entirely of in-scale tones and those sequences that included some out-of-scale tones, but also sensitivity to the difference between sequences with implied regular-harmony and those with irregular-harmony. These results show that training in Western music environment, familiar to the average children, LeNTS acquired a valid scale schema in an early stage of learning and following this, with more exposure, this network also later acquired a valid harmony schema. The present results show that the learning trajectory of LeNTS is consistent with that generally observed in musically westernized children (Trainor and Trehub, 1994; Corrigall and Trainor, 2010, 2014).

What suggestions for the acquisition process of listeners’ tonal schema do these simulation results offer? One possible suggestion concerns functional changes of neural circuitry in conjunction with progress in acquiring a tonal schema. Although the network architecture was fixed during training, small and incremental changes occurred in connection weights. As a result, LeNTS was able to simulate the learning changes of tonal schema in musically westernized listeners. Thus, it is possible to draw inferences about changes in neural circuitry as several researchers of connectionist models have done (e.g., McClelland, 1989). That is, in contrast to the idea of stage-like transitions involving abrupt changes during development as some postulate (e.g., Piaget, 1954), the current simulation depicts changes in the neural circuitry underlying tonal processing that do not change drastically at certain points in development. Rather, tonal schema learning is incremental, changing gradually as a function of exposure to music. Indeed, this possibility is consistent with findings compiled in various neuroimaging studies. One line of neuroimaging studies, based on data from young children to adults, consistently focuses on Early Right Anterior Negativity (ERAN) brain activity (Koelsch, 2012) and it shows that ERAN reflects the high-order neural processing based on tonal schema. Although limited, the available data provided by these studies suggests that ERAN responses are incrementally modulated during development. Studies of Western adult listeners show that ERAN has a negative polarity, maximal amplitude values over the frontal leads (inferior frontal gyrus; Maess et al., 2001), and a peak latency of 125–250 ms following a tonal deviant (Koelsch et al., 2000; Koelsch and Jentschke, 2010). In older children, ERAN responses share the common spatial (e.g., inferior frontal gyrus; Koelsch et al., 2005) and temporal properties (a peak latency of 200–250 ms) with those of adults, although the ERAN was a bilateral scalp distribution (Jentschke and Koelsch, 2009). In younger children, ERAN responses involves the frontal brain regions as in older children and adults, although they have a slightly increased peak latency (300–350 ms) compared to older children and adults (Jentschke et al., 2014), as well as a positive polarity perhaps indicative of an overall weaker brain signal (Corrigall and Trainor, 2014). To summarize, adults, older children, and younger children share a common characteristic that the ERAN (tonal processing) involves activity of the frontal regions such as inferior frontal cortex. In addition, although the strength and temporal properties of ERAN are slightly different among the three age groups, the differences seem to be under certain constraints. In light of this, it is unlikely that spatial, strength, and temporal properties of neural circuitry of tonal processing undergo a drastic change at some point in development. Rather, as in the present simulation, learning is evident in small and incremental changes in listeners’ neural circuitry of tonal organization as they gain experience with music.

Another possible suggestion concerns factors contributing to the speed of schema acquisition during exposure to music. In general, it is assumed that age-dependent changes (a term used here to denote changes scheduled by an innate timetable, e.g., maturation) and experience-dependent changes are reflected in the speed of acquisition process. In this study, learning of LeNTS depended only on the total number of epochs of tone sequence input data. Nevertheless, the tonal schema acquisition process of LeNTS was consistent with the accumulated evidence of tonal schema acquisition by children in a Western music culture (Trainor and Trehub, 1992, 1994; Corrigall and Trainor, 2010). Therefore, based on such simulations, we can infer that children’s tonal schema acquisition does not depend strictly on maturational factors, but that the speed of acquisition is primarily determined by the amount of exposure to music experienced during childhood. Recently, Trainor and her colleagues have reported that active musical participation in infancy enhances the acquisition of culture-specific musical schema (Gerry et al., 2012) and that children who participate in music or dance classes are more sensitive to tonal deviations than children who do not, even among children equated for age (Corrigall and Trainor, 2014). Such individual differences observed on the speed of tonal schema acquisition can be explained by the concept of experience-dependent learning.

The other suggestion concerns the learning principles of the brain mechanism involving tonal processing. In this study, we designed the hidden layers of LeNTS to not be bound by culture-specific properties. Nonetheless, the results reveal that LeNTS was able to acquire harmonic schema, which is specific to listeners of the Western music, from mere exposure to Western melodic sequences. Considering this, it is possible that if we retain these hidden layers but change the melodic training materials to reflect non-Western music, LeNTS may also be capable of acquiring the relevant, but culture-specific, tonal schema. The idea is supported by results of Matsunaga et al. (2013), which reports that LeNTS is able to extract the relevant tonal schema through mere exposure to traditional Japanese melodies. It therefore seems likely that the computational basis underlying developmental changes in the neural circuitry of tonal processing are fundamentally equivalent across listeners of different cultures, even if the resulting acquired tonal schemas are varied with the exposure to culture-specific music.

The simulations of the present study represent the first steps toward understanding the tonal schema acquisition process of listeners. However, in order to pursue this, the following three points must be considered. First, a closer examination of training methods for the network would be useful. In this study, each melodic training material appeared once in 1 epoch from the beginning of the training. This means that LeNTS was simultaneously trained with the large number of melodies from the beginning. However, it is unlikely that children begin this process by learning the large number of melodies in their culture. In reality, the amount of melodies that children encounter in daily life increases incrementally. To mimic this incremental increase, data showing a clearer picture of listener music environments in each development period (infant, younger children, older children, adults) are necessary. To our best knowledge, such data has not been reported, and this should be addressed in the future studies.

The second point is that the present study excluded the processing of metrical organization. In most connectionist models (e.g., Bharucha, 1987; Tillmann et al., 2000) including us, the input layer processed only tone chroma information. Nonetheless, the existing musical pieces include not only information of tone chroma but also information of time length (i.e., intervals between onsets of each tone; i.e., abbreviated as onset-to-onset intervals “IOIs”). Some studies indicate that the metrical organization influences tonal organization (Abe and Okada, 2004), although note that the opposite pattern has also been reported (Prince et al., 2009). If we construct a network in which the input layer is able to process tone chroma information as well as that of the information of intervals between tone onsets, the network should be able to deal with musical input data that is more realistic. Consequently, the trained network may explain the broader parts of music processing in the human brain.

The third point is that our current model was not equipped with the mechanisms related to the lower-level processing of pitch perception which underlies the higher-level processing of tonal perception. The reason for it is that it is well-known psychological fact that tonal perception is based on a symbolic sequence of tone chroma categories of constituent notes of a melody (e.g., Krumhansl, 1990; Koelsch, 2012). Nonetheless, if we extend our model to being capable of treating real sounds as the input data, the model should incorporate the lower-level pitch processing stage prior to the tone chroma processing stage.

## Conclusion

We constructed a connectionist network (LeNTS) that succeeded in fulfilling three basic requirements where previous networks have failed. We found that when LeNTS was trained with Western music, it acquired the Western culture-specific tonal schema in a learning process similar to that of children in the general population. In the past 20 years, there has been an increase in the number of neuroimaging studies, and the basic properties of brain activities involving tonal organization processing have been gradually clarified (Koelsch, 2012). Recently, brain activities of tonal processing have been investigated from the viewpoints of development differences (Koelsch et al., 2005), expertise differences (Oechslin et al., 2012), and culture differences (Matsunaga et al., 2012, 2014), and findings from these studies have expanded and deepened the understanding of tonal processing in the brain. However, neuroimaging findings only describe the static characteristics of the human brain. In other words, the neuroimaging findings have difficulties in providing suggestions for dynamic computational characteristics. To further understand tonal processing in the human brain, it is necessary to integrate findings from both neuroimaging studies and computational modeling studies.

## Conflict of Interest Statement

The authors declare that the research was conducted in the absence of any commercial or financial relationships that could be construed as a potential conflict of interest.

## Footnotes

1 Although musical systems used in Indian and Arabic–Persian culture are based on 22 possible intervals per octave, in practice such microtones function as “ornaments” and the basic structure of the inclusive scale is essentially the same as that of the Western 12-interval chromatic scale (Burns, 1999).

2 In general, listeners have difficulty identifying a stable key for a tone sequence including out-of-scale tones (e.g., Hoshino and Abe, 1984). Therefore, caution is warranted when interpreting that C major was inferred as the correct key for all tone sequences of group (c). However in this study we decided to provisionally follow results of Cuddy et al. (1981).

## Appendix

In the preliminary simulation, we prepared three different networks that shared the same input and output layers. This was also true of the network prepared in the main simulation, the input layer represented 12 tone chromas, and the output layer represented 24 keys. The main difference among the three networks was the number of hidden layers. The three networks each had one, two, and three hidden layers, respectively (hereafter, we called them LeNTS 1, LeNTS 2, and LeNTS 3, respectively). For each network, five different runs were carried out; the network was initialized with random connection weights in each run. Training melody materials were identical to those used in the main simulation. The three networks were trained for 50,000 epochs. After training, the networks were tested with the novel set of tone sequences (which differed from those used in the main simulation). In testing, a musical key, which corresponded to the output unit with the highest activation, was interpreted as “the identified key” of the network. Then, keys identified by the networks were compared to those perceived by most musically trained listeners, and the agreement rates were calculated. A one-way ANOVA revealed that the agreement rates differed among the three networks [*F*(2,12) = 5.40, *p* < 0.05]. The agreement rate for LeNTS 1 was significantly lower than that for LeNTS 3 (*p* < 0.05), but there were not significant differences in a comparison between LeNTS 1 and LeNTS 2, nor were there in another comparison between LeNTS 2 and LeNTS 3. These results mean that LeNTS 1 and LeNTS 3 significantly differed but LeNTS 2 and LeNTS 3 did not. According to the scientific explanation of parsimonious principle, simpler and fewer assumptions are preferable to more complex ones. Following the parsimonious principle, we selected two layers, not three layers, in the hidden positions of the network of the main simulation.
